# Dietary niche partitioning and convergent gut microbiota in sympatric *Vespa*

**DOI:** 10.3389/fmicb.2026.1804127

**Published:** 2026-06-02

**Authors:** Caojinge Li, Tantan Gao, Lifeng Meng, Jiaxing Huang

**Affiliations:** 1 State Key Laboratory of Resource Insects, Institute of Apicultural Research, Chinese Academy of Agricultural Sciences, Beijing, China; 2 College of Bioscience and Resource Environment, Beijing University of Agriculture, Beijing, China

**Keywords:** 16S rRNA, DNA metabarcoding, feeding habit, gut microbiota, sympatric, *Vespa*

## Abstract

Gut microbiota plays a crucial role in nutrition acquisition and environmental adaptation in social hornets (*Vespa* spp.), yet how host phylogeny correlates with dietary niche partitioning and gut microbial community in sympatric species remain poorly understood. Here, we combined mitochondrial *COI* phylogenetics, multi-locus metabarcoding (*COI*/ *trnL*-P6) dietary profiling, and 16S rRNA-based gut microbiota characterization across four sympatric *Vespa* species. *COI* phylogeny revealed two distinct clades: (*Vespa tropica* + *V. basalis*) and (*V. velutina* + *V. bicolor*). Dietary preferences and gut microbiota composition matched host phylogeny: the first clade was characterized by Fagales consumption and variable insect prey (*V. basalis*: Coleoptera; *V. tropica*: Hymenoptera) and Pseudomonadota dominance, the second specialized in Hymenoptera/Ericales and Bacillota. This pattern suggests that host evolutionary history may shape microbial community and dietary niches. Functional prediction suggested enhanced secondary metabolite biosynthesis in the *V. tropica* clade versus upregulated carbohydrate metabolism in the *V. velutina* clade, highlighting the influence of food niches on gut microbiota function. Within clades, fine-scale dietary partitioning (e.g., *V. velutina* prefers *Apis*, while *V. bicolor* specializing on Megachilidae bees) minimizes interspecific competition, revealing the coexistence strategies of sympatric *Vespa* species. Our findings suggest a potential interaction between host phylogeny and dietary adaptation in shaping gut microbiota, which requires further validation with larger sample sizes.

## 1 Introduction

Social hornets (*Vespa* spp.) are apex predators and pivotal ecological regulators, profoundly influencing insect population dynamics and floral resource utilization (Otis et al., 2023). Their ecological impacts include direct suppression of insect populations via predation and the pathogen transmission (Dalmon et al., 2019; Rojas-Nossa et al., 2023), invasive species like *Vespa velutina* disrupt local ecosystems and agricultural productivity by preying on honey bees and other beneficial insects (Pedersen et al., 2025), which have attracted growing interest. Their foraging strategies are fundamental to their ecological success and impact (Ueno, 2015; Chen et al., 2017). These strategies are shaped by resource availability, intrinsic physiological states such as prior experience, learning and memory capacity, and interspecific competition (Mandal and Brahma, 2019; Shibata and Kudo, 2020; Patterson and Drury, 2023). However, the molecular mechanisms underlying niche partitioning among sympatric species remain unclear.

*Vespa* species exhibit broad dietary habits, acquiring protein through predation on insects and small vertebrates, and obtaining carbohydrates from nectar, fruits, and plant sap (Richter, 2000). While early studies relied on limited morphological identification and field observations (Matsuura and Sakagami, 1973). Recently, stable-isotope analysis (δ^13^C and δ^15^N for trophic positioning) reveals that *Vespa* species are highly flexible predators. With the advancement of DNA metabarcoding, significant progress has been achieved in investigating the feeding habits of *Vespa* species (Rome et al., 2021; Do et al., 2023). For example, studies on 1,500 larval gut samples of *V. velutina* from Europe demonstrated that this species exhibits a diverse dietary composition, including honey bees (Pedersen et al., 2025), and *Vespa mandarinia* preys on over 56 species across multiple phyla (Wilson et al., 2023). However, comparative analyses of the feeding habits of sympatric *Vespa* species remain scarce, which limits our understanding of how diet mediates their coexistence and fitness.

The gut microbiota serves as a critical interface linking diet to host physiology, contributing to nutrition, digestion, immunity, and fitness in social insects (Ruppert et al., 2019; Liberti et al., 2024; Jing et al., 2020). Unlike the gut microbiomes of corbiculate bees (e.g., honey bees and bumble bees), which harbor ancient, coevolved symbionts for pollen or nectar digestion (Koch et al., 2013; Li et al., 2023), *Vespa* gut microbiomes are simple but functionally specific (Suenami et al., 2019; Zhang et al., 2022; Do et al., 2023), dominated by transient bacteria acquired from their trophic niche. For instance, *Vespa mandarinia* and *Vespa simillima* harbor only 7–8 core operational taxonomic units (OTUs) (Suenami et al., 2019), and *V. velutina*’s microbiota varies with foraging locations (Zhang et al., 2022; Do et al., 2023). These patterns suggest that diet-microbiota interactions may drive host ecological adaptation to diverse dietary niches via flexible gut microbiota; yet, how host evolutionary history constrains these relationships remains unexamined in *Vespa* (Zhang and Zheng, 2022).

Dietary specialization is a key determinant of gut microbiota composition, with herbivores and predators harboring distinct bacterial guilds responsible for fiber degradation or proteolysis (Vernier et al., 2020; Yin et al., 2024). In *Vespa*, however, the tripartite interplay between phylogenetic relatedness, dietary niche, and microbial assembly has never been comprehensively examined, leaving unanswered questions: whether closely related species share similar diets and/or microbial communities, and whether this “gene-diet-microbiota” axis explains their ecological adaptation. Addressing these questions is critical to unraveling the mechanisms of niche partitioning in social hornets.

In this study, we employed a multifaceted approach combining mitochondrial *COI* gene sequencing, DNA metabarcoding (using *COI* and *trn*L-P6) and 16S rRNA gene amplicon sequencing to investigate the evolutionary relationships, dietary ecology, and gut microbiomes of four sympatric *Vespa* species (*Vespa tropica*, *Vespa basalis*, *V. velutina*, and *Vespa bicolor*) native to East and Southeast Asia, predominantly southern China and adjacent regions (Otis et al., 2023). We aimed to (1) resolve their phylogenetic relationships using *COI* sequences; (2) compare dietary niches and identify resource partitioning; (3) characterize the composition and function of gut microbiota; and (4) explore the potential role of host evolutionary history in shaping diet-microbiota interactions, and ecological adaptation. This integrative framework aims to reveal how *Vespa* species coexist through phylogeny-linked dietary and microbial adaptation, providing insight into their role in ecosystem stability and relevant management strategies.

## 2 Materials and methods

### 2.1 Samples and dissection

**Figure 1:**
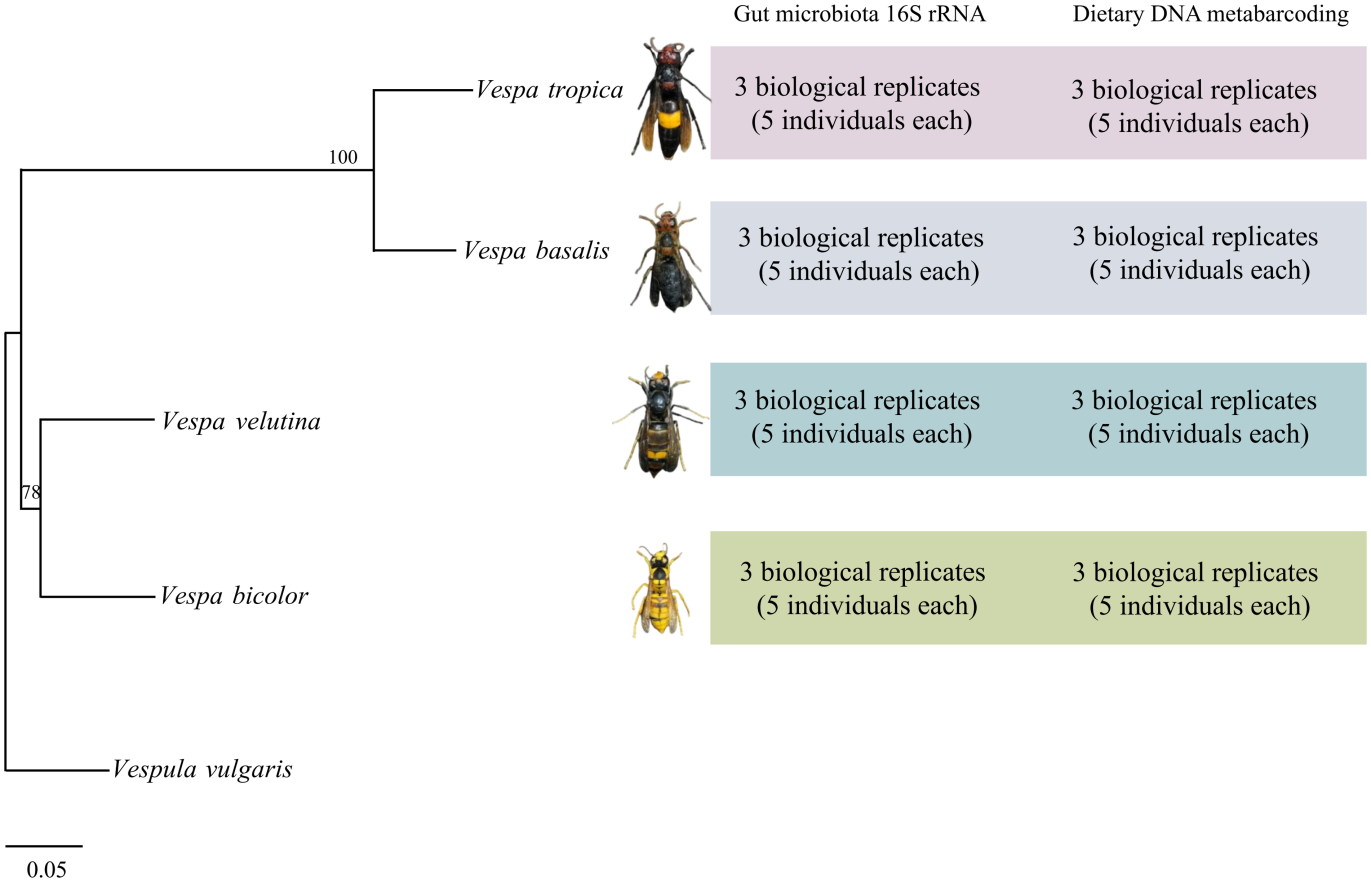
Phylogenetic tree of four *Vespa* species based on COI gene sequences. Scale bar: 0.05 nucleotide substitutions per site, numbers at nodes indicate bootstrap support value from 1,000 replicate; Outgroup: *V. vulgaris*. All hornet photographs were taken by the authors and are original images without copyright restrictions.

Workers of *V. tropica* (*n* = 33), *V. basalis* (*n* = 36), *V. velutina* (*n* = 60) and *V. bicolor* (*n* = 46) were collected from apiaries at the Cangyuan Experimental Station of the Institute of Apiculture Research, Chinese Academy of Agricultural Sciences (23°18′27.4″N, 99°07′26.5″E; elevation 1,304 m) from July to early November 2024, using sterile entomological nets (60 mesh, 30 cm diameter, and 50 cm depth). Specimens were immediately transferred into 50 mL sterile conical tubes containing RNA stabilization solution (RNAwait, Beijing Biotopped) at a 1:50 (W/V) ratio. Following 24 h fixation at 4 °C, samples were cryopreserved in vapor-phase liquid nitrogen prior to storage at −80 °C until dissection. Worker specimens of the *Vespa* species were dissected under aseptic conditions on ice using sterile scissors and forceps. Of these collected individuals, 15 workers per species were used for dissection. Gut contents from five individuals were combined to form a single sample, with three biological replicates per species and stored at −80 °C for subsequent dietary and microbiota analysis (Figure 1).

### 2.2 Host phylogenetic analysis based on *COI* barcoding

To investigate the phylogeny of four *Vespa* species, *COI*-based phylogenetic analyses were conducted using established barcoding protocols. Genomic DNA (gDNA) was extracted from legs of individual *Vespa* species using the DNeasy Blood & Tissue Kit (Qiagen, Hilden, Germany) according to the manufacturer’s instructions. The mitochondrial *COI* gene was amplified via PCR with Lepidoptera-specific primers LepF1 (5′-ATTCAACCAATCATAAAGATA TTGG-3′) and LepR1 (5′-TAAACTTCTGGATGTCCAAAAAATCA-3′) under the following conditions: 95 °C for 5 min, 35 cycles of 94 °C for 30s, 50 °C for 30s, 72 °C for 1 min, and a final extension at 72 °C for 5 min. PCR products were Sanger-sequenced bidirectionally on an ABI 3730xl DNA Analyzer (Applied Biosystems, Waltham, United States). Raw sequences were assembled using SeqMan (DNASTAR Lasergene Suite v6), and primer regions (LepF1/LepR1) along with low-quality bases (Phred score < 20) were trimmed manually in BioEdit v7.0.5.3. Consensus sequences (length: ~650 bp) were aligned with ClustalW using default parameters, and a neighbor-joining (NJ) phylogenetic tree was constructed in MEGA11 (Tamura et al., 2021) using the Kimura 2-parameter (K2P) model (selected via ModelTest) and 1,000 bootstrap replicates. The phylogenetic tree was rooted with *V. vulgaris* as the outgroup (GenBank accession number: KJ415829.1).

### 2.3 DNA extraction and quality control

For dietary and microbiota analyses, 50 mg of wet weight of pooled gut contents was homogenized separately from each of the four *Vespa* species (*V. tropica*, *V. basalis*, *V. velutina*, *V. bicolor*) in 1 mL of SLX-Mlus Buffer (Qiagen), using a Tissue Lyser II (45 Hz, 250 s). Genomic DNA (gDNA) was extracted using the Omega DNA Extraction Kit (Cat. No. D4015-02, Omega Bio-tek, Norcross, United States) according to the manufacturer’s instructions, yielding high-quality DNA suitable for subsequent analyses. The concentration and purity of extracted DNA were quantified using a NanoDrop 2000 spectrophotometer (Thermo Fisher Scientific, United States) by measuring the absorbance ratios at 260/280 nm. DNA integrity was evaluated via 1% agarose gel electrophoresis (5 V/cm, 20 min), intact genomic DNA exhibited a single clear banding without smearing. All qualified DNA samples were divided into two aliquots for parallel dietary metabarcoding and 16S rRNA gene sequencing assays.

### 2.4 Dietary DNA metabarcoding experiment

To characterize the dual dietary requirements of *Vespa* species, (protein acquisition for development and carbohydrate intake for sustenance), a cross-kingdom metabarcoding approach was employed. Insect prey composition was determined by amplifying the mitochondrial cytochrome c oxidase subunit I (*COI*) gene using degenerate primers NoPlantF_270 (5′-RGCHTTYCCHCGWAT AAAYAAYATAAG-3′) and ml*COI*intR_W (5’-GRGGRTA WACWGTTCAWCCWGTNCC-3′) (Krehenwinkel et al., 2022), In parallel, plant dietary components were traced by targeteing the chloroplast *trn*L-P6 intron with primers *trn*L g/h (5′-GGG CAATCCTG AGCCAA-3′/5′-CCATTGAGTCTCTGCACCTAT C-3′) (Kartzinel et al., 2019). Triplicate 20 μL PCR reactions were performed for each target locus using FastPfu DNA Polymerase (TransGen Biotech, Beijing, China), with each sample labeled with a unique 8-base barcode sequence to enable multiplexing. PCR amplicons were separated by 2% agarose gels electrophoresis, and target bands (COI: ~270 bp; *trnL*-P6: ~150 bp) were excised and purified using the AxyPrep DNA Gel Extraction Kit (Axygen Biosciences, United States) according to the manufacturer’s instructions. Purified PCR products were quantified by a Qubit®3.0 Fluorometer (Life Invitrogen, United States). Equal molar amounts of amplicons (24 unique barcodes per pool) were combined to construct sequencing libraries. Briefly, pooled DNA was fragmented by ultrasonication, followed by duplex unwinding and removal of uncircularized DNA molecules to generated standard sequencing libraries using the Illumina TruSeq® Nano DNA LT Kit. Libraries were sequenced on the MGI-G99 platform (Shanghai BIOZERON Biotech) in paired-end 300 bp (PE300) mode according to the standard protocols. Sequencing data were processed as described in Section 2.6, with raw fastq files subjected to unified quality control and analysis.

### 2.5 16S rRNA gene amplification and sequencing for gut microbiota

A total of 12 genomic DNA samples (3 biological replicates per species from *V. tropica*, *V. basalis*, *V. velutina*, and *V. bicolor*) were subjected to 16S rRNA gene amplicon sequencing at Shanghai BIOZERON Biotech. Co., Ltd. The V4 hypervariable region was amplified using barcoded primers 515F 5′-GTGCCAGCMGCCGC GGTAA-3′ and 806R 5′-GGACTACHVGGGTWTCTAAT-3′. Triplicate 20 μL PCR reaction contained 4 μL of 5 × FastPfu Buffer, 2 μL of 2.5 mM dNTPs, 0.8 μL of each primer (5 μM), 0.4 μL of FastPfu Polymerase, and 10 ng of template DNA. Thermocycling conditions were as follows: initial denaturation at 95 °C for 2 min, followed by 25 cycles at 95 °C for 30 s, 55 °C for 30 s, and 72 °C for 30 s, and a final extension at 72 °C for 5 min. Amplicons were gel-purified (AxyPrep DNA Gel Extraction Kit, Axygen Biosciences, Union City, CA, U. S.), quantified via Qubit®3.0 Fluorometer (≥5 ng/μL, Life Invitrogen, United States), and normalized before pooling 24 uniquely barcoded amplicons. Library construction, ultrasonication fragmentation, and PE300 sequencing on the MGI-G99 platform were performed following the identical protocols described in Section 2.4. with all procedures complying with the Minimum Information about any (x) Sequence (MIxS) standards for reliable downstream microbiota analysis (Yilmaz et al., 2011).

### 2.6 Unified sequencing data preprocessing pipeline

Raw fastq files from Sections 2.4 (dietary metabarcoding) and 2.5 (16S rRNA sequencing) were preprocessed. Trimmomatic (Bolger et al., 2014) and in-house scripts were used for demultiplexing and filtering. Filtering criteria were as follows: (i) 300 bp reads were truncated at any site with an average Phred quality score <20 over a 10 bp sliding window, and reads <50 bp post-trimming were discarded; (ii) exact barcode matching, ≤2 primer mismatches, and removal of reads with ambiguous characters; 2-nucleotide mismatch in primer matching, containing ambiguous characters; (iii) paired-end reads were merged only if the overlap length ≥10 bp and the maximum mismatch rate in the overlap region ≤ 20%, with unassembled reads discarded.

OTUs were clustered with 97% similarity using UPARSE (Edgar, 2013), and chimeric sequences were removed using UCHIME via a hybrid strategy [*de novo* detection combined with reference-based alignment against the Gold database (Edgar et al., 2011)]. For dietary data (Section 2.4), sequence were annotated against the NCBI NT database [UCLUST algorithm, 80% confidence (Kim and Chun, 2014)]. For microbiota data (Section 2.5), annotation was performed against the Silva SSU138.2 database (Quast et al., 2012) using the same UCLUST parameters.

### 2.7 Statistical and functional analysis of dietary and microbiota data

#### 2.7.1 Dietary data analysis

Linear discriminant analysis effect size (LEfSe; Segata et al., 2011) was used to identify dietary biomarkers for the four *Vespa* species: a Kruskal-Wallis sum-rank test (*p* < 0.05) examined interspecific taxon differences, followed by LDA (log10 > 2) to determine the effect size of distinctively abundant taxa (Ijaz et al., 2018; Feng et al., 2024).

#### 2.7.2 Gut microbiota analysis

Downstream 16S rRNA analysis utilized the CFViSA platform (Wang N. et al., 2024) and the vegan package in R-forge. Rarefaction analysis and diversity indices (Simpson and Shannon) (Wang N. et al., 2024) were calculated using Mothur v.1.21.1 (Schloss et al., 2009). Beta diversity was analyzed using Bray-Curtis distance matrices, with the principal co-ordinates analysis (PCoA) for visualization (Xiong et al., 2020). LEfSe was employed to identify microbiota biomarkers, follwing the same statistical workflow as dietary data analysis (Segata et al., 2011).

Functional prediction was performed via PICRUSt2 (Langille et al., 2013): OTU tables were normalized by 16S rRNA copy number, converted to BIOM format, and mapped to the KEGG database for pathway annotation. Low-abundance features (relative abundance < 0.01%) were filtered, data normalized to relative abundances, and functional profiles analyzed at different KEGG levels. All data were visualized using R v3.1.1 (pheatmap package) and Cytoscape.^1^

### 2.8 Correlation analysis between dietary component and gut microbiota

Spearman’s rank correlation coefficient was used to assess the relationships between dietary components (insect and plant taxa) and gut microbiota, as proportional diet data and bacterial relative abundances were non-normally distributed with extreme values, violating Pearson correlation’s normality assumptions (Supplementary Table S1). The analysis included three types of variables (dietary insect taxa, plant taxa, and gut microbiota), all expressed as with-sample relative abundances. To reduce data dimensionality and improve interpretability, representative taxa were selected for correlation analysis and heatmap visualization based on their occurrence frequency across all samples. Occurrence frequency was defined as the proportion of samples in which a given taxon was detected, regardless of its abundance level. Specifically, the top 10 insect taxa, top 10 plant taxa, and top 20 gut microbiota taxa with the highest occurrence frequency were retained. Prior to correlation analysis, relative abundances were first calculated within each sample based on all valid reads from that sample. The selection of representative taxa described was performed only after the original within-sample relative abundances were computed; no renormalization or recalculation of relative abundances was conducted among the selected subset of taxa. Spearman’s rank correlation coefficients (r) were calculated pairwise between dietary taxa (insect and plant) and gut bacterial genera. To control for potential false discoveries from multiple comparisons, *p*-values were adjusted using the Benjamini–Hochberg (BH) false discovery rate (FDR) correction. Correlations were considered statistically significant at FDR < 0.05. Only significant correlations were visualized in the heatmap, whereas non-significant correlations were masked to enhance clarity. All statistical analyses were performed using the Biozeron Cloud Platform (Wang K. et al., 2024).

## 3 Results

### 3.1 Phylogenetic relationships of hornets based on *COI* gene sequences

The neighbor-joining (NJ) tree shows two monophyletic clades within the *Vespa* genus: one clade comprising *V.* tropica and *V. basalis*, and a second clade containing *V. velutina* and *V. bicolor* (Figure 1). All interspecific nodes received strong bootstrap support The outgroup *V. vulgaris* formed a distinct basal branch.

### 3.2 Dietary diversity of *Vespa* species revealed by *COI* and *trn*L-P6 gene sequencing

#### 3.2.1 Overview of the *Vespa* diet

For dietary analysis of *Vespa*, 1,431,324 mitochondrial *COI* gene and 1,395,232 chloroplast *trn*L-P6 locus sequences were obtained using two primers set (Supplementary Table S2). A total of 455 insect-derived Operational Taxonomic Units (OTUs) were identified with the mitochondrial *COI* marker, and 6,279 plant-derived OTUs were determined using the targeted chloroplast *trnL*-P6 locus across four *Vespa* species (*V. tropica*, *V. basalis*, *V. velutina,* and *V. bicolor*).

#### 3.2.2 Insect diversity and composition in the diet of *Vespa* species

**Figure 2:**
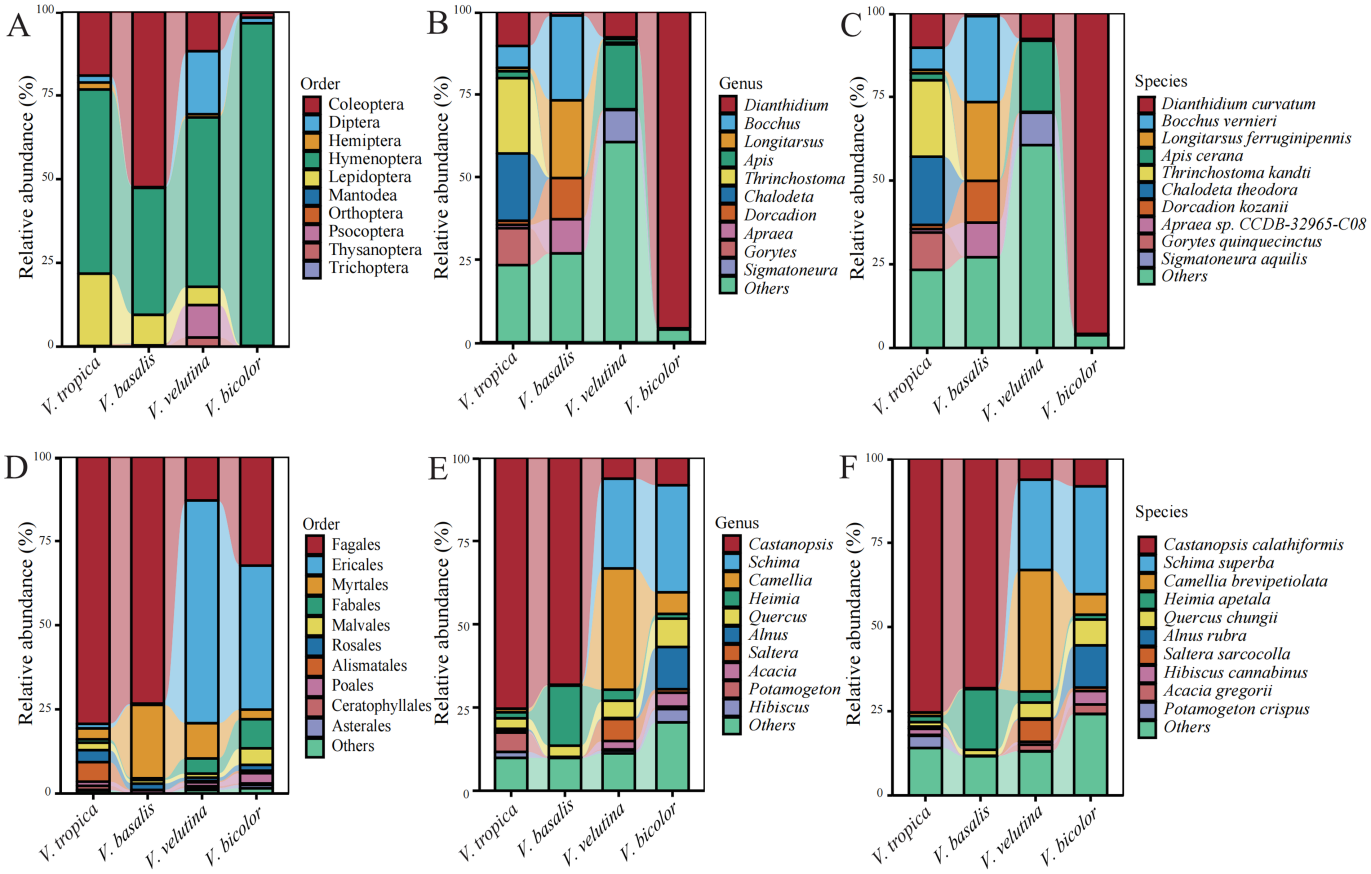
The dietary composition of *Vespa tropica*, *Vespa basalis*, *Vespa velutina*, and *Vespa bicolor*. Insect-derived dietary components at different taxonomic levels: **(A)** Order, **(B)** genus, and **(C)** species. Plant-derived dietary components at different taxonomic levels: **(D)** Order, **(E)** genus, **(F)** species. Taxa with a relative abundance of less than 1% across all samples were collectively labeled as “Others”.

**Figure 3:**
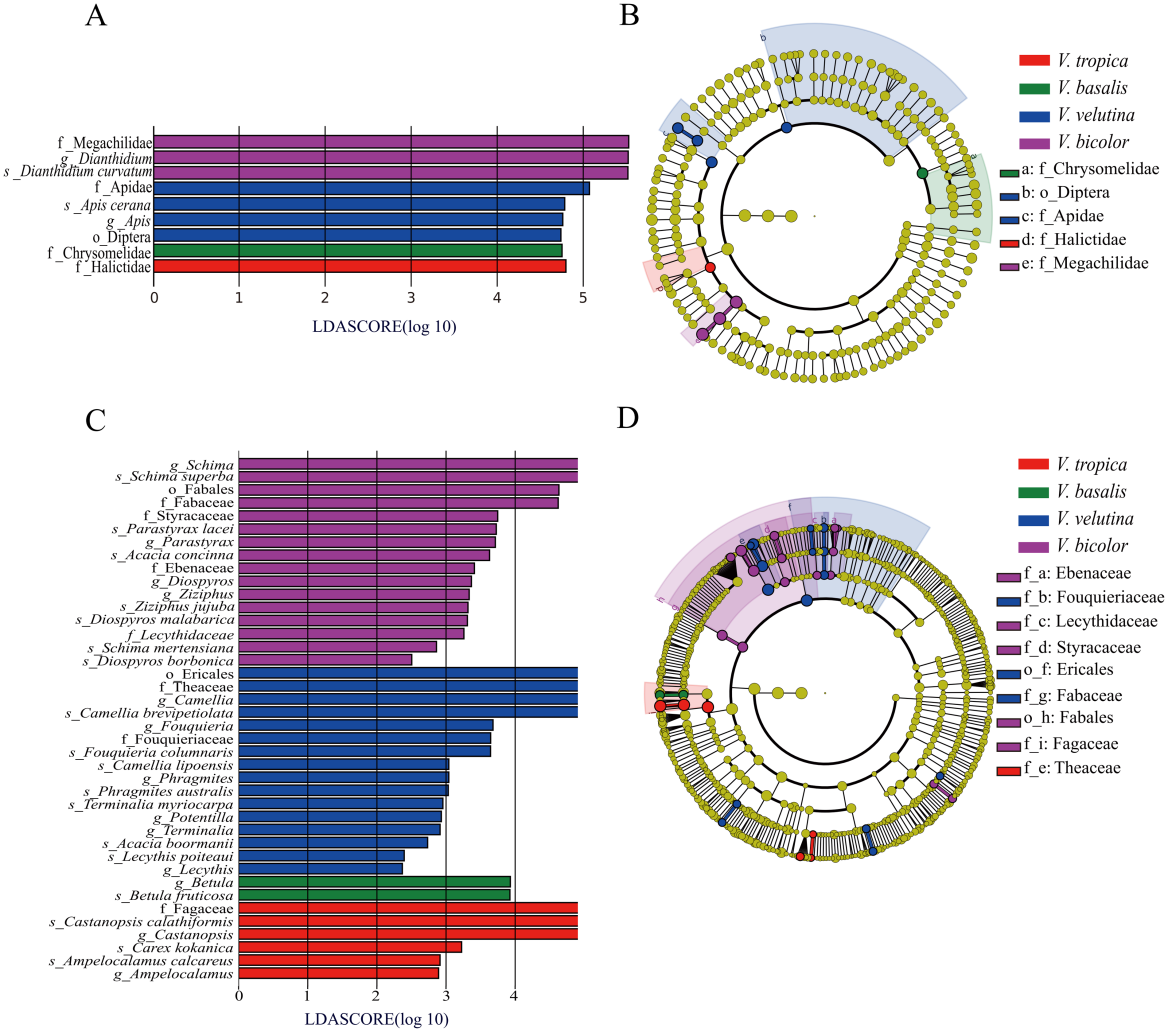
Species-specific dietary biomarkers of four sympatric *Vespa* species identified via LEfSe analysis. **(A)** Log_10_-transformed LDA scores for insect OTUs. Letters preceding OTUs denote taxonomic levels (*p* = phylum, *c* = class, *o* = order, *f* = family, and *g* = genus). **(B)** The cladogram visualizes the hierarchical distribution of insect biomarkers significantly enriched in each *Vespa* species. Yellow nodes denote taxa with no significant biomarker signals, while colored nodes indicate taxa that are significant biomarkers. **(C)** Log_10_-transformed LDA scores for OTUs of plants consumed by four *Vespa* species. Letters preceding OTUs denote taxonomic levels (*p* = phylum, *c* = class, *o* = order, *f* = family, and *g* = genus). **(D)** The cladogram displays the phylogenetic hierarchy of plant biomarkers enriched in individual hornet species.

Dietary analysis of four *Vespa* species showed consistent predation preferences across insect taxa. All species commonly prey on Hymenoptera insects, followed by Coleoptera, Lepidoptera, and Diptera (Figure 2A). Notably, *V. tropica* consumed Hymenoptera (54.96%), Lepidoptera (21.08%), and Coleoptera (19.08%), whereas *V. basalis* favored Coleoptera (52.37%). *V. velutina* preyed on Hymenoptera (50.55%), Diptera (18.82%), and Psocoptera (9.68%), while *V. bicolor* almost exclusively preyed on Hymenoptera (98.38%) (Figure 2A). Significant divergence emerged at the family, genus and species levels, revealing a specialized feeding strategy distinct from that of co-occurring *Vespa* species. Specifically, *V. tropica* uniquely consumed *Thrinchostoma kandti* (22.83%) and *Gorytes quinquecinctus* (11.15%) (Figures 2B,C), with a pronounced enrichment of Halictidae (Figure 3A). *V. basalis* primarily consumed Chrysomelidae (Figure 3A), and key species were *Bocchus vernieri* (25.65%) and *Longitarsus ferruginipennis* (23.53%) (Figures 2B,C). *V. velutina* exhibited the broadest dietary niche (Shannon/Simpson indices) across 50 genera (Supplementary Table S3), with specific enrichment in Apidae (e.g., *Apis cerana* 21.24%) (Figures 2A, 3A,B). *V. bicolor* exclusively preyed on *Dianthidium* (95.80%) of the family Megachilidae (Figure 2B), which was further confirmed by LefSe analysis showing enrichment of the genus *Dianthidium* and species *Dianthidium curvatum* (Figures 3A,B).

#### 3.2.3 Plant diversity and composition in the diet of *Vespa* species

Dietary analysis of the four *Vespa* species revealed significant trophic divergence into two distinct groups. *V. tropica* and *V. basalis* preferentially fed on plants within the Fagales order, specifically *Castanopsis* species such as *Castanopsis calathiformis*, *V. tropica* consumed Fagales (79.31%), *Castanopsis calathiformis* (75.34%) across taxonomic levels (Figures 2D–F), which was confirmed by LEfSe analysis (Figures 3C,D); *V. basalis* fed on Fagales (73.29%), chiefly *Castanopsis, C. calathiformis* (68.15%) (Figures 2D–F). Conversely, *V. bicolor* and *V. velutina* primarily targeted plants of the Ericales order, particularly *Schima* species including *Schima superba*, for *V. velutina* specialized in Ericales (66.15%) with auxiliary Fagales (12.95%), dominated by *Camellia brevipetiolata* (36.04%) and *Schima superba* (26.80%) (Figures 2D–F); this was further confirmed by LEfSe enrichment of Ericales, the family Theaceae, the genus *Camellia*, and the species *C. brevipetiotata* (Figures 3C,D); *V. bicolor* consuming Ericales (42.77%), Fagales (32.31%), Fabales (8.64%), and Poales (3.56%), with key species *Schima superba* (31.99%) and *Alnus rubra* (12.52%) (Figures 2D–F), LEfSe also revealed enrichment for Fabales, families Fagaceae, genera *Schima*, and species like *S. superba* (Figures 3C,D). Furthermore, *V. bicolor* and *V. velutina* exhibited a broader plant dietary spectrum, and *V. bicolor* exhibited the broadest dietary diversity (Shannon/Simpson indices; 218 genera in 61 families), and *V. velutina* had the highest prey abundance (199 genera from 66 families) (Supplementary Table S4).

### 3.3 Gut bacterial communities

#### 3.3.1 Gut microbiota composition across four *Vespa* species

427,958 high-quality paired-end sequence reads were generated through 16S rRNA gene sequencing. After quality filtering and chimera removal, 1,393 bacterial OTUs were obtained. Phylogenetic classification revealed a hierarchical taxonomic distribution across 24 bacterial phyla, 44 classes, 111 orders, 192 families, 419 genera, and 101 species, with an average effective read length of 252.9 bp (Supplementary Table S5).

#### 3.3.2 Distribution characteristics of gut microbiota of *Vespa* species

**Figure 4:**
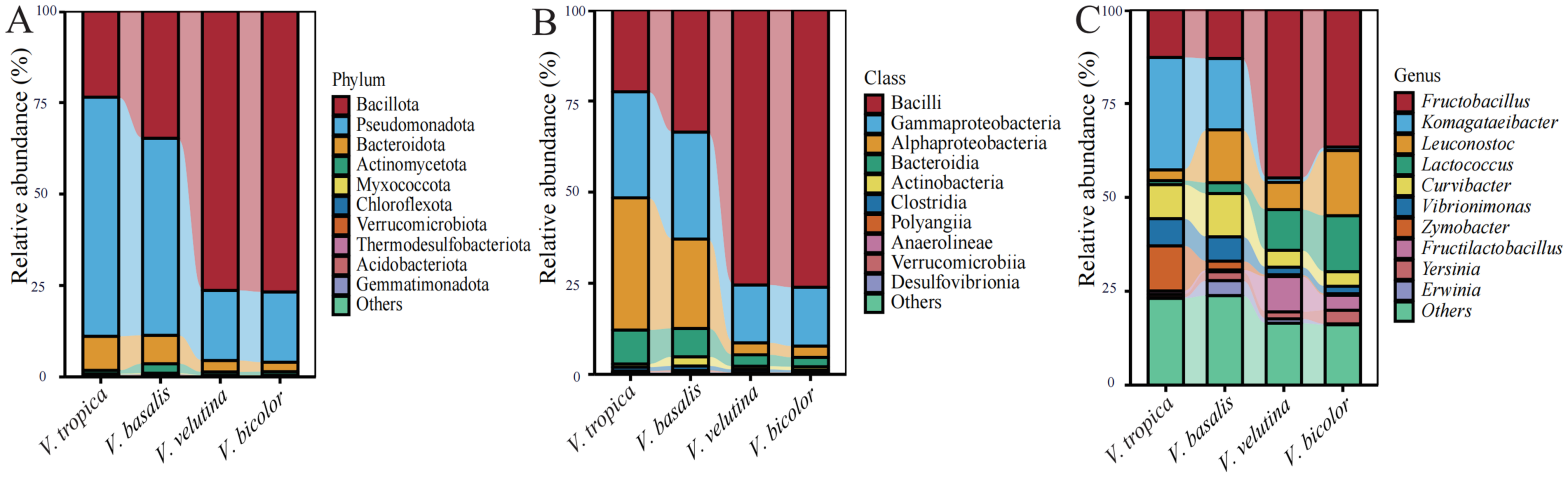
Gut microbiota composition of *Vespa tropica*, *Vespa basalis*, *Vespa velutina*, and *Vespa bicolor* at different taxonomic levels: **(A)** Order, **(B)** Genus, and **(C)** Species. Taxa with a relative abundance of less than 1% across all samples were collectively labeled as others.

Analysis of gut microbiota composition revealed that the four *Vespa* species exhibited similar profiles at the phylum and class levels, primarily dominated by Bacillota, Pseudomonadota, Bacteroidota, and Actinomycetota. However, a bipartite divergence was observed, with Pseudomonadota significantly enriched in *V. tropica* (65.35%) and *V. basalis* (53.86%), whereas Bacillota dominated in *V. velutina* (76.33%) and *V. bicolor* (76.75%) (Figure 4A). At the class level, Bacilli, Gammaproteobacteria and Alphaproteobacteria constituted the predominant intestinal bacterial groups. *V. tropica* and *V. basalis* exhibited consistent profiles, with higher relative abundances of Alphaproteobacteria and Gammaproteobacteria, in comparison, *V. velutina* and *V. bicolor* exhibited identical dominant bacterial compositions, with Bacilli and Gammaproteobacteria being the most abundant (Figure 4B). At the Genus level, the core microbiota of four *Vespa* species gut comprised 6 to 9 genera, including *Fructobacillus*, *Komagataeibacter*, *Leuconostoc*, *Curvibacter*, etc. (Figure 4C). However, significant variations in bacterial species composition and relative abundances were observed across host species: *Komagataeibacter*, *Curvibacter*, *Vibrionimonas*, and *Zymobacter* were dominant in *V. tropica* and *V. basalis*, while *Fructobacillus*, *Lactococcus*, *Leuconostoc*, and *Fructilactobacillus* were the predominant in *V. velutina* and *V. bicolor* (Figure 4C).

#### 3.3.3 Diversity comparison of the gut microbiome of four *Vespas* species

**Figure 5:**
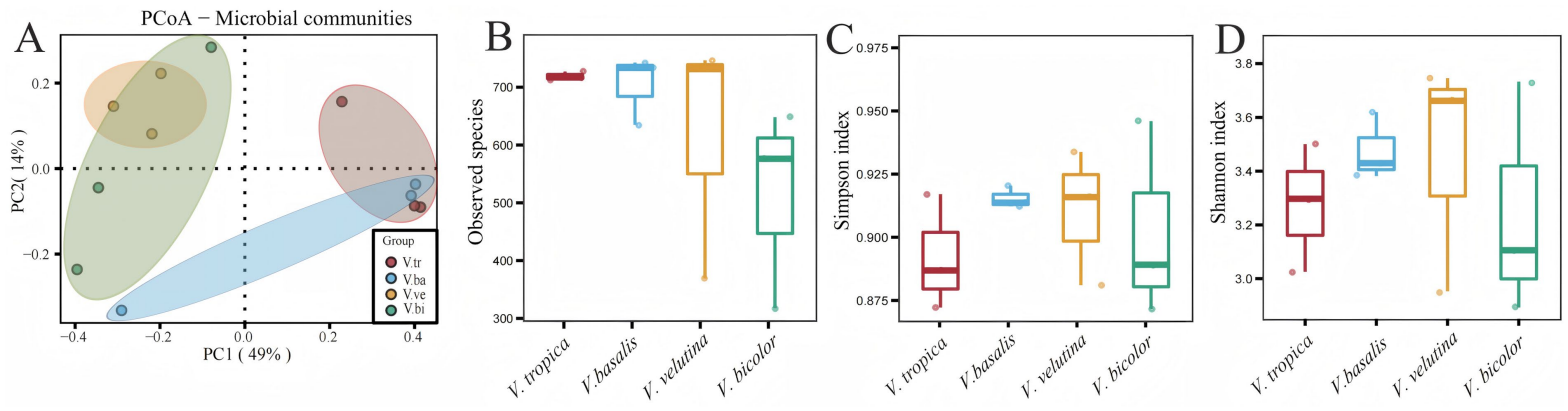
Beta diversity of gut microbial in *Vespa tropica*, *Vespa basalis*, *Vespa velutina*, and *Vespa bicolor*. **(A)** Principal coordinates analysis (PCoA) based on unweighted Bray-Curtis distance of the four *Vespa* species (*V. tr* = *V. tropica*; *V. ba* = *V. basalis*; *V. ve* = *V. velutina*; *V. bi* = *V. bicolor*); Alpha diversity of gut microbial in *Vespa tropica*, *Vespa basalis*, *Vespa velutina*, and *Vespa bicolor*. Examination of gut bacterial communities in the four *Vespa* species using **(B)** species richness observed; **(C)** Simpson index **(D)** Shannon index. No significant differences in bacterial diversity were observed.

Principal coordinates analysis (PCoA) based on Bray-Curtis distances revealed distinct clustering of bacterial communities along the first two components (PCoA1 and PCoA2), which collectively explained 49 and 14% of the total variance at the OTUs level, respectively. *V. tropica* and *V. basalis* were clustered together, while *V. velutina* and *V. bicolor* formed distinct clusters (Figure 5A).

Alpha diversity metrics, including observed species richness (Figure 5B), Shannon evenness index (Figure 5C), and inverse Simpson index (Figure 5D), revealed no significant differences (*p* > 0.05) among the four *Vespa* species.

**Figure 6:**
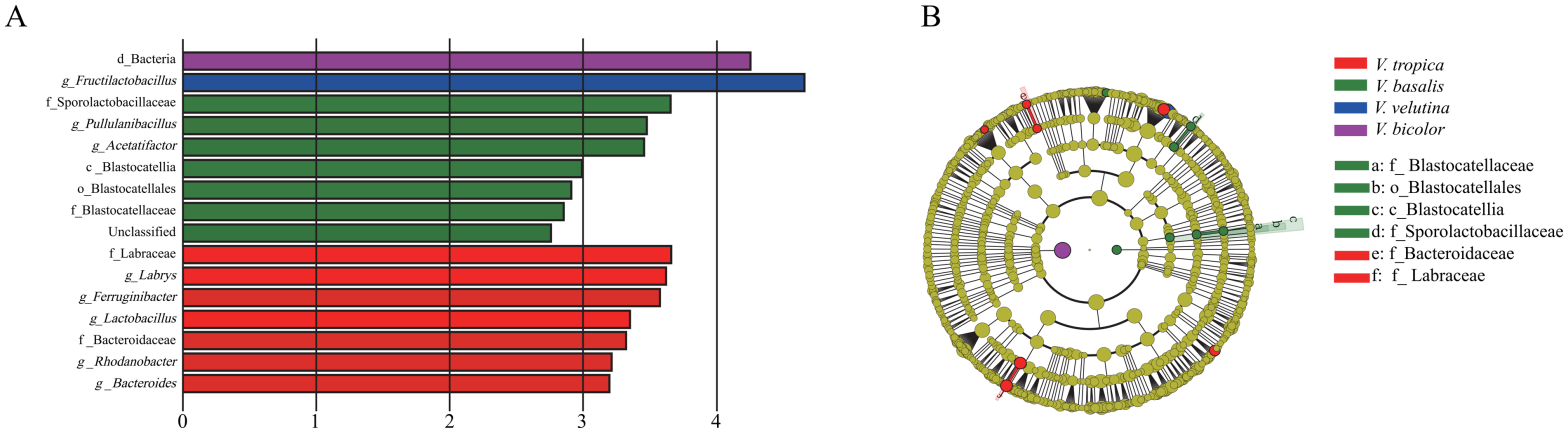
Species-specific gut microbial biomarkers in *Vespa tropica*, *Vespa basalis*, *Vespa velutina*, and *Vespa bicolor* identified through LEfSe analysis. **(A)** Log10-transformed LDA scores showing differentially abundant bacterial taxa enriched in each *Vespa* species. Letters preceding taxa denote taxonomic levels (p = phylum, c = class, o = order, f = family, g = genus). **(B)** The cladogram visualizes the hierarchical phylogenetic distribution of gut microbial biomarkers significantly enriched in individual hornet species. Yellow nodes denote taxa with no significant biomarker signals, while colored nodes indicate taxa that are significant biomarkers.

LEfSe identified differential taxonomic enrichment at multiple phylogenetic levels. *V. tropica* was enriched in the family Labraceae and Bacteroidaceae, and the genera *Labrys*, *Ferruginibacter*, *Lactobacillus*, and *Bacteroides*. *V. basalis* was dominant in the class Blastocatellia, the order Blastocatellales, the family Sporolactobacillaceae and Blastocatellaceae, the genera *Pullulanibacillus* and *Acetatifactor*. *V. velutina* was specifically enriched in the genus *Fructilactobacillus*. *V. bicolor* did not show any taxa uniquely identified as biomarkers (Figure 6A). The cladogram further visualized these lineage-specific signatures, highlighting differential representation of 1 order, 1 class, and 4 families (Figure 6B).

#### 3.3.4 Function profiling of *Vespa* species gut microbiota via PICRUSt2 prediction

**Figure 7:**
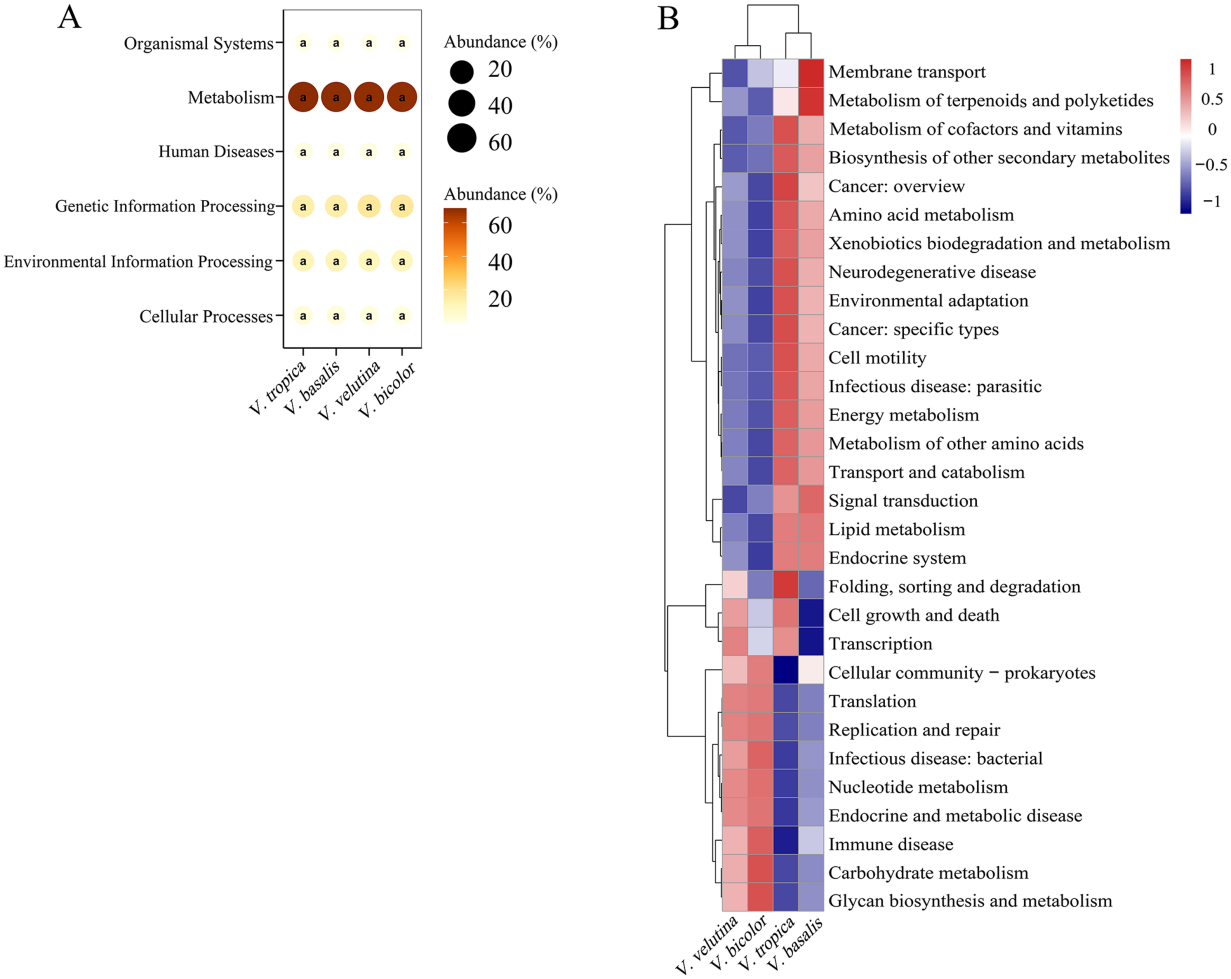
Functional pathways of gut microbes predicted by PICRUSt2 in *Vespa tropica*, *Vespa basalis*, *Vespa velutina*, and *Vespa bicolor*. **(A)** Relative abundances of predicted KEGG pathways at level 1. Bubble size and color both represent the relative abundance (%) of each functional category in each *Vespa* species. **(B)** Heatmap of predicted KEGG pathways at level 2. Values were normalized by *Z*-score across *Vespa* species for each pathway. Red and blue colors indicate pathways with higher and lower relative abundance compared with the mean level of each pathway across species, respectively. Hierarchical clustering was performed based on Euclidean distance.

PICRUSt2-based functional prediction of gut microbiota revealed no significant differences in KEGG Level 1 pathways among the four *Vespa* species, with primary functional enrichment concentrated in metabolic function, accounting for 68% of the predicted function (Figure 7A, Supplementary Table S6).

Divergent enrichment emerged at Level 2: biosynthesis of secondary metabolites and amino acid metabolism were primarily elevated in *V. tropica* and *V. basalis*. In contrast, carbohydrate metabolism and glycan biosynthesis and metabolism constituted the most enriched function groups in *V. velutina* and *V. bicolor* (Figure 7B, Supplementary Table S7).

### 3.4 Correlation analysis between insects, plants, and gut microbiota

**Figure 8:**
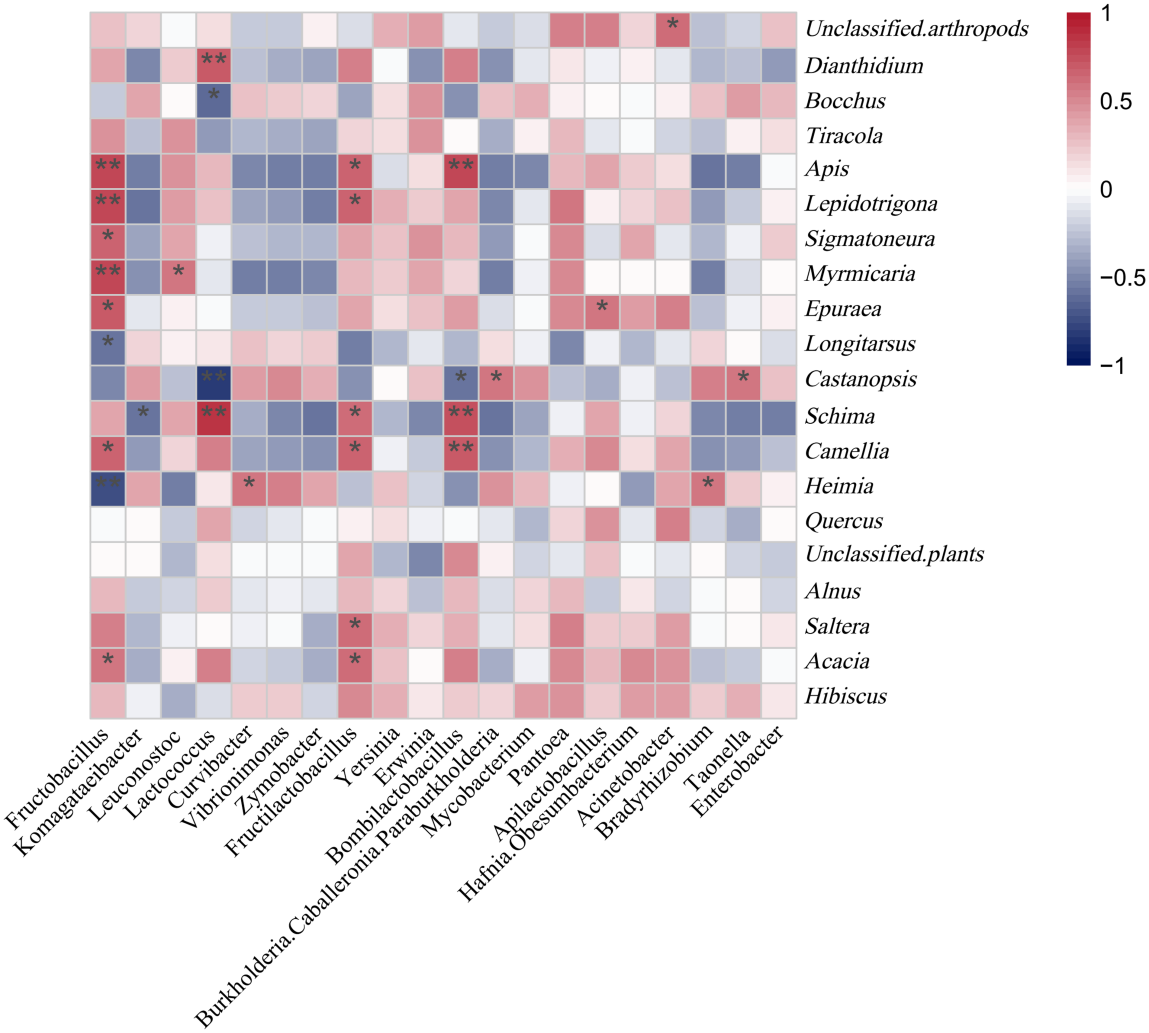
Heatmap illustrating the genus level associations among the top 10 insect genera, top 10 plants, and top 20 gut microbial genera. Colors intensity ranges from blue (negative correlation) to red (positive correlation). Significance was determined using Benjamini–Hochberg (BH)-adjusted *p*-values, with *p < 0.05 and **p < 0.01 and ***p < 0.001.

Spearman correlation analysis between dietary components (insect prey or plant resources) and gut microbial taxa revealed two clusters of significant diet-microbe interactions. *Lactococcus* positively correlated with *Dianthidium* and *Schima; Fructobacillus* with *Apis, Lepidotrigona* and *Camellia*; *Fructilactobacillus* with *Schima, Camellia, Apis* and *Lepidotrigon*; *Bombilactobacillus* with *Apis*, *Schima and Camellia.* Conversely, *Lactococcus* exhibited a negative correlation with *Bocchus* and *Castanopsis* (all *p* < 0.05, significance tiers are indicated in Figure 8).

## 4 Discussion

Based on an integrated analysis of *COI* phylogeny, dietary metabarcoding, and gut microbiome profiling of four sympatric *Vespa* species, a general congruence was observed between host phylogenetic relationships, dietary niche differentiation, and gut microbial community structure: closely related species exhibited similar dietary preferences and microbial assemblages, and this phylogenetic signal in diet and microbiota facilitating the coexistence of sympatric hornets through fine-scale resource partitioning. COI phylogeny showed two distinct clades: *V. tropica* clustered with *V. basalis*, while *V. velutina* formed a sister clade with *V. bicolor*, reflecting their evolutionary divergence. This clade-based divergence generally aligned with dietary and microbial differentiation, with clade I (*V. tropica* + *V. basalis*) preferentially consuming Fagales and showing divergent insect prey preferences, and being enriched in Pseudomonadota related to secondary metabolite degradation. Clade II (*V. velutina* + *V. bicolor*) specialized on Hymenoptera/Ericales and was enriched in Bacillota, which is associated with carbohydrate metabolism. Notably, fine-scale dietary partitioning occurs within both clades: within Clade I, *V. basalis* mainly preys on Coleoptera while *V. tropica* primarily consumes Hymenoptera; within Clade II, *V. velutina* prefers *Apis* whereas *V. bicolor* specializes on Megachilidae bees. This dual-level differentiation (broad clade-level conservatism plus fine-scale within-clade divergence) minimizes interspecific competition and promotes stable coexistence. This study confirmed that phylogenetic relatedness structures ecological adaptation via diet-microbiota interactions, establishes phylogeny-diet-microbiota linkage as a key coexistence mechanism and provides a metabarcoding framework for understanding *Vespa* nutritional ecology and ecosystem impact.

### 4.1 Dietary specialization and gut microbiota differentiation in social *Vespa* linked to their phylogenetic relationships

The intricate relationship among host evolution, diet, and gut microbiota in *Vespa* species reveals a phylogenetically linked pattern of ecological adaptation, characterized by distinct dietary specialization and microbial divergence between closely related species pairs. This finding supports the view that host evolutionary history and diet jointly shape gut microbial community assembly in wild animals (Youngblut et al., 2019; Kang et al., 2022). Host phylogenetic divergence drives coordinated dietary specialization and gut microbial differentiation, which further mediates ecological adaptation in sympatric hornets. This clear differentiation underscores the pivotal role of host diet in shaping gut microbiome composition, and corroborates the profound influence of host evolutionary history on this divergence (Neha et al., 2025), and implies that host intrinsic factors, shaped by shared ancestry, may initially define the permissible dietary niche, which in turn filters and structures the gut microbial community (Mallott and Amato, 2021). The observed functional divergence-with enhanced biosynthesis of secondary metabolites in the *V. tropica* clade versus upregulated carbohydrate metabolism in the *V. velutina* clade, further underscores that dietary specialization, mediated by phylogeny, drives functional adaptation of the gut microbiota to optimize nutrient extraction from distinct food resources (Zhang et al., 2023). Such a pattern is consistent with the concept of “ecological fitting,” in which host lineages utilize ancestral traits to colonize new niches, with the gut microbiota rapidly assembling to support the utilization of specialized diets (Neha et al., 2025).

Notably, we addressed the potential concern of “secondary predation” (i.e., whether the detected species DNA originated from the prey’s diet rather than the hornets’ active foraging). For instance, The diet of *V. bicolor* was dominated almost entirely by Megachilidae (*Dianthidium curvatum*, 95.80%), while a small proportion of Fabaceae plants (*Acacia*, 4.18%) was detected in its plant-derived sequences. Considering that Megachilidae are essential pollinators of Fabaceae, this association may suggest the occurrence of “secondary predation” (Udayakumar and Shivalingaswamy, 2022). However, multiple lines of evidence support the interpretation that plant DNA originated from active foraging by *vespa*: (i) If plant DNA were mainly derived from prey, other species that prey on sympatric Megachilidae (e.g., *V. tropica*) would also yield Fabaceae, which was not the case; (ii) Only gut contents were analyzed, minimizing contamination from exogenous materials; (iii) Although *V. basalis* preys on Coleoptera (e.g., *Longitarsus*) that feed on Fabaceae, no Fabaceae was detected in its plant-derived lineages (Prathapan, 2016). Importantly, adult *Vespa* species cannot survive without nectar (Bouchebti et al., 2022), indicating that the correspondence between insect prey and plant lineages likely reflects the coupling between foraging breadth, habitat use, and resource availability in *Vespa*, rather than simple secondary predation. While secondary predation cannot be entirely excluded, its effect is likely minimal and does not affect the conclusion that *Vespa* species actively forage on plant resources. In addition, the high proportion of Megachilidae (*D. curvatum*) in the diet of *V. bicolor* may reflect seasonal prey specialization, local population outbreaks of the prey species, or biases associated with metabarcoding primer amplification and reference database limitations. Although this pattern has not been independently verified, it only affects the relative abundance of a single prey group and thus has a negligible impact on the overall conclusion regarding dietary divergence among the four *Vespa* species.

### 4.2 Dietary divergence reduces interspecific resource competition among four sympatric *Vespa* species

Classical ecological theory posits that resource partitioning promotes species coexistence by reducing interspecific competition (Armstrong and McGehee, 1980), and dietary partitioning represents a crucial ecological axis for sympatric species to avoid competition (Greene et al., 2020). A study on four sympatric grasshopper species of the subfamily Gomphocerinae in subalpine meadows of the French Alps indicates that restricted diet differentiation facilitates coexistence by reducing competition (Ibanez et al., 2013). It has been reported that four invasive wasp species (two *Vespula* and two *Polistes*) on Ahuahu Island, New Zealand, avoid competition through niche shifts (Schmack et al., 2021). In this study, sympatric *Vespa* species with close genetic relationships avoid competition through fine-scale dietary differentiation, as supported by LEfSe analysis (Figure 3) and Spearman correlation analysis (Figure 8), insect prey resources show clear divergence among closely related species, whereas plant resource utilization is highly conserved and strongly mirrors the host phylogenetic clade pattern.

These statistically supported patterns demonstrate that *Vespa* species partition limited and contested insect prey resources, while converging on abundant and widely available plant resources, thereby minimizing competitive pressure. This strategy is ecologically stable because plant-derived carbohydrates (e.g., nectar and sap) are generally abundant and less likely to trigger intense competition. When resources are plentiful, species can occupy shared nutritional niches while avoiding direct conflict over limited prey (Balfour et al., 2015). Such hierarchical resource use therefore supports the long-term stable coexistence of closely related *Vespa* species within the same habitat.

### 4.3 Food preference as a key driver of gut microbiota functional variation

Dietary differences shape both the composition and function of the gut microbiota in *Vespa* species. Nutritional content varies among food resources: Coleopteran insects are characterized by high protein content (Hasnan et al., 2023), whereas Hymenopteran insectsexhibit higher glycogen content (Ying and Xiao, 1999). Fagales species are tannin-rich, and Ericales species are nectar-rich (Zhou et al., 2012; Jiang et al., 2025). In the present study, we found that *V. basalis* and *V. tropica* which prefer protein-rich Coleoptera and tannin-rich Fagales, harbored significantly higher abundances of Gammaproteobacteria and *Komagataeibacter* genus known for efficient protein degradation. In contrast, *V. velutina* and *V. bicolor*, which feed on glysogen-rich Hymenotera (*Apis*, *Dianthidium*) and nectar-rich Theaceae (*Schima*, *Camellia*), exhibited higher abundance of lactic acid bacteria (LAB; including *Fructilactobacillus*, *Leuconostoc*, *Lactococcus*, *Fructobacillus*) and Bacillus-related taxa, groups specialized in carbohydrate metabolism. Spearman correlation analyses validated these associations: LAB (e.g., *Lactococcus*, *Fructobacillus*, *Fructilactobacillus, Bombilactobacillus*) were positively correlated with glycogen-rich insects (*Apis* and *Lepidotrigona*) and nectar-rich plants (*Camellia and Schima*), whereas *Lactococcus* was negatively associated with tannin-rich *Castanopsis*. These results indicate that LAB abundance is positively associated with dietary sugars and glycogen (Takatani and Endo, 2021), while tannin-rich plants may suppress LAB colonization (Zhu et al., 2022; Chen et al., 2023).

Functional prediction via PICRUSt2 showed that *V. tropica* and *V. basalis* exhibit higher proportions of pathway related to the biosynthesis of other secondary metabolites and amino acid metabolism. In comparison, *V. velutina* and *V. bicolor* showed elevated carbohydrate metabolism and glycan biosynthesis. These patterns suggest a close linkage between gut microbial function and host nutritional ecology, facilitating adaptation to available food resources (Felice et al., 2019), and highlight the strong influence of food composition on the structure and function of the gut microbiota.

### 4.4 Dual ecological impacts of sympatric predation: pollinator decline counterbalanced by pest control mediated through gut microbiome adaptation

Early studies recognized *Vespa* as important predatory insects due to their strong hunting ability and high foraging efficiency (Chi et al., 2018). The invasive *V. velutina* has caused significant declines in honey bee populations, prompting a reassessment of the ecological role of *Vespa* species (Laurino et al., 2019; Diéguez-Antón et al., 2025). Based on LEfSe analysis in this study, the four sympatric *Vespa* species exhibit marked dietary differences. *V. basalis* primarily preys on Chrysomelidae (*Longitarsus ferruginipennis*), indicating a potential role in controlling herbivorous pests (Kelley and Dobler, 2011). In contrast, the other three species exhibit selective predation on members of Apoidea, including Halictidae, Apidae, and Dianthidium, which are important pollinators of natural vegetation and crops (Hristov et al., 2020; Hinson et al., 2024). Predation on these pollinating insects may impair pollination services, reduce plant reproductive success, and threaten the stability of ecosystem functions.

Notably, *V. velutina* feeds not only on bees but also on Diptera pollinators and several Psocoptera decomposers. Dipterans similarly play crucial roles in pollination, while Psocopterans contribute to the decomposition of organic matter and nutrient cycling (Doyle et al., 2020; Raguso, 2020). Predation on these functionally important groups may lead to reductions in species diversity, disrupt energy flow and nutrient cycling, and potentially trigger cascading effects on ecosystem stability (Mills et al., 1992). Among the four *Vespa* species, *V. velutina* exhibits the broadest insect dietary niche and harbors gut microbiota enriched in functions related to replication and repair, cell growth and death (Figure 7B). These microbial functions are closely associated with maintaining genome stability, cellular homeostasis, and stress tolerance in bacteria, which may enhance the stability and functional resilience of the gut microbial community under fluctuating dietary and environmental conditions. Such improved microbial functional stability could indirectly benefit the host by supporting consistent nutrient supply and physiological homeostasis, thereby improving adaptability to environmental fluctuations and contributing to its invasion success and ecological destructiveness. Therefore, future ecological management should carefully consider the “dual ecological effects” of *Vespa* species: harnessing their potential benefits in pest control while mitigating their negative impacts on pollinator communities and ecosystem functions to develop more balanced and sustainable management strategies.

### 4.5 Limitations

This study has several limitations that should be acknowledged. First, pooled gut samples were used due to the small size of *Vespa* guts. Individual samples do not yield sufficient DNA for sequencing and library preparation, making pooling a necessary and widely adopted strategy in small insect and birds microbiome research (Dada et al., 2021; Garcia-Mazcorro et al., 2021). Although pooling limits the assessment of inter-individual variance, the substantial variation among pooled samples (Figure 5) primarily reflects natural biological heterogeneity in wild populations, such as differences in microhabitat and foraging history, rather than technical inconsistency. Second, functional prediction of the gut microbiome via PICRUSt2 have inherent limitations: inference based on 16S rRNA gene sequences may be inaccurate due to strain-level genome variation and incomplete reference databases for non-human microbiotas (Douglas et al., 2020). Future studies could use individual samples (with improved sequencing sensitivity) and shotgun metagenomic sequencing to validate the present findings. Additionally, the small number of sympatric species examined in this study limits the generalizability of our findings, and larger sample sizes in future research will help strengthen the associations observed between phylogeny, diet, and gut microbiota.

## Data Availability

All raw sequencing data generated in this study have been deposited in the NCBI Sequence Read Archive (SRA) under BioProject accession number PRJNA1372002 . The COI sequences have been deposited in GenBank under accession numbers PZ211678–PZ211681, https://dataview.ncbi.nlm.nih.gov/object/PRJNA1372002?reviewer=j6425d435da8q95sod48hvofmu.

## Footnotes

1
http://www.cytoscape.org
