# Supplementary material for: Dietary niche partitioning and convergent gut microbiota in sympatric Vespa
Source: Front Microbiol. 2026 Jun 2;17:1804127. doi: 10.3389/fmicb.2026.1804127 (PMC13269277; doi:10.3389/fmicb.2026.1804127)
Supplement: Supplementary file 1 [file Table_1.docx]

**Supplemental Tables**

**Table S1. Normality test results (Shapiro–Wilk test) of variables in diet–gut microbiota correlation analyses.**

| Variable | Shapiro-Wilk test | |
| --- | --- | --- |
|  | W statistic | *p* |
| Unclassified-arthropods | 0.750 | 0.003** |
| *Dianthidium* | 0.602 | 0.000** |
| *Bocchus* | 0.331 | 0.000** |
| *Apis* | 0.527 | 0.000** |
| *Tiracola* | 0.328 | 0.000** |
| *Lepidotrigona* | 0.506 | 0.000** |
| *Sigmatoneura* | 0.338 | 0.000** |
| *Myrmicaria* | 0.336 | 0.000** |
| *Epuraea* | 0.388 | 0.000** |
| *Longitarsus* | 0.451 | 0.000** |
| *Castanopsis* | 0.845 | 0.032* |
| *Schima* | 0.696 | 0.001** |
| *Camellia* | 0.617 | 0.000** |
| *Heimia* | 0.594 | 0.000** |
| *Quercus* | 0.659 | 0.000** |
| *Unclassified-plants* | 0.681 | 0.001** |
| *Alnus* | 0.365 | 0.000** |
| *Saltera* | 0.581 | 0.000** |
| *Acacia* | 0.770 | 0.004** |
| *Hibiscus* | 0.610 | 0.000** |
| *Fructobacillus* | 0.879 | 0.085 |
| *Komagataeibacter* | 0.766 | 0.004** |
| *Leuconostoc* | 0.682 | 0.001** |
| *Lactococcus* | 0.648 | 0.000** |
| *Curvibacter* | 0.757 | 0.003** |
| *Vibrionimonas* | 0.748 | 0.003** |
| *Zymobacter* | 0.450 | 0.000** |
| *Fructilactobacillus* | 0.703 | 0.001** |
| *Yersinia* | 0.708 | 0.001** |
| *Erwinia* | 0.502 | 0.000** |
| *Bombilactobacillus* | 0.629 | 0.000** |
| *Burkholderia-Caballeronia-Paraburkholderia* | 0.807 | 0.011* |
| *Mycobacterium* | 0.516 | 0.000** |
| *Pantoea* | 0.757 | 0.003** |
| *Apilactobacillus* | 0.357 | 0.000** |
| *Hafnia-Obesumbacterium* | 0.437 | 0.000** |
| *Acinetobacter* | 0.451 | 0.000** |
| *Bradyrhizobium* | 0.789 | 0.007** |
| *Taonella* | 0.793 | 0.008** |
| *Enterobacter* | 0.720 | 0.001** |

Note: * p<0.05*,* ** p<0.01.*p*-values were calculated via the Shapiro–Wilk test to assess the normality of the data distribution. A *p*-value < 0.05 indicates a significant deviation from normality.Tables were ordered by the occurrence frequency of taxa associated with *Vespa* diet and gut microbiota, including the top ten insect genera and the top ten plant genera in the diet, and the top twenty bacterial genera detected in the gut microbiota.

**Table S2.** Statistical summary of amplicon sequencing data for dietary analysis using insect- and plant-spcific primers

| Primer | Samples  Average Length(bp) | Sequences | Bases(bp) |
| --- | --- | --- | --- |
| NoPlantF270-mlCOIintRW *V. tropica*1 | 111396 | 7130574 | 64.01 |
| NoPlantF270-mlCOIintRW *V. tropica*2 | 111907 | 7162482 | 64.00 |
| NoPlantF270-mlCOIintRW *V. tropica*3 | 127588 | 8166262 | 64.00 |
| NoPlantF270-mlCOIintRW *V. basalis*1 | 126385 | 8087065 | 63.99 |
| NoPlantF270-mlCOIintRW *V. basalis*2 | 123703 | 7916238 | 63.99 |
| NoPlantF270-mlCOIintRW *V. basalis*3 | 113363 | 7255140 | 64.00 |
| NoPlantF270-mlCOIintRW *V. velutina*1 | 117680 | 7530682 | 63.99 |
| NoPlantF270-mlCOIintRW *V. velutina*2 | 119860 | 7672068 | 64.01 |
| NoPlantF270-mlCOIintRW *V. velutina*3 | 120656 | 7723298 | 64.01 |
| NoPlantF270-mlCOIintRW *V. bicolo*1 | 112858 | 7222509 | 64.00 |
| NoPlantF270-mlCOIintRW *V. bicolo*2 | 120169 | 7690574 | 64.00 |
| NoPlantF270-mlCOIintRW *V. bicolo*3 | 125759 | 8048371 | 64.00 |
| trnLg-trnLh *V. tropica*1 | 99031 | 6415790 | 64.79 |
| trnLg-trnLh *V. tropica*2 | 100255 | 6599005 | 65.82 |
| trnLg-trnLh *V. tropica*3 | 121051 | 7636135 | 63.08 |
| trnLg-trnLh *V. basalis*1 | 125436 | 7877207 | 62.80 |
| trnLg-trnLh *V. basalis*2 | 115857 | 7316614 | 63.15 |
| trnLg-trnLh *V. basalis*3 | 125019 | 7862792 | 62.89 |
| trnLg-trnLh *V. velutina*1 | 126501 | 6825846 | 53.96 |
| trnLg-trnLh *V. velutina*2 | 120017 | 6513244 | 54.27 |
| trnLg-trnLh *V. velutina*3 | 114667 | 6222624 | 54.27 |
| trnLg-trnLh *V. bicolo*1 | 98210 | 6308969 | 64.24 |
| trnLg-trnLh *V. bicolo*2 | 128145 | 6940929 | 54.16 |
| trnLg-trnLh *V. bicolo*3 | 121082 | 6438455 | 53.17 |

**Table S3.** Diversity of insect diet in four *Vespa* species.

|  | ***V. tropica*** | ***V. basalis*** | ***V. velutina*** | ***V. bicolor*** |
| --- | --- | --- | --- | --- |
| **Observed_species** | 72 | 64 | 129 | 90 |
| **Shannon** | 8.736553104 | 5.415643544 | 8.802208086 | 7.952019738 |
| **Simpson** | 2.777272456 | 2.184459471 | 2.697390313 | 2.7060615 |

Notes: Observed value was the total total number of identified OTU species.The Shannon and Simpson indices were used to evaluate community diversity: (high Shannon index+ low Simpson index) = high diversity without a dominant species; (low Shannon index + high Simpson index) = Low diversity dominated by a single species.

**Table S4.** Diversity of plant diet in four *Vespa* species.

|  | ***V. tropica*** | ***V. basalis*** | ***V. velutina*** | ***V. bicolor*** |
| --- | --- | --- | --- | --- |
| **Observed_species** | 4581 | 4460 | 4841 | 4494 |
| **Shannon** | 8.252591173 | 7.897997072 | 9.002756341 | 9.92989118 |
| **Simpson** | 1.95801314 | 2.024601757 | 2.371457405 | 2.55679976 |

Notes: Observed value was the total number of identified OTU species. The Shannon and Simpson indices were used to evaluate community diversity: (high Shannon index+ low Simpson index) = high diversity without a dominant species; (low Shannon index + high Simpson index) = Low diversity dominated by a single species.

**Table S5.** Sequencing output and read length statistics of 16S rRNA gene (V4 region) amplicons from four *Vespa* species generated using primers 515F–806R

| Primer | Samples | Sequences | Bases(bp) | AverageLength(bp) |
| --- | --- | --- | --- | --- |
| 515F-806R | *V. tropica*1 | 36264 | 9173725 | 252.97 |
| 515F-806R | *V. tropica*2 | 37231 | 9418233 | 252.97 |
| 515F-806R | *V. tropica*3 | 33148 | 8385135 | 252.96 |
| 515F-806R | *V. basalis*1 | 33664 | 8516393 | 252.98 |
| 515F-806R | *V. basalis*2 | 38518 | 9741365 | 252.9 |
| 515F-806R | *V. basalis*3 | 35857 | 9070627 | 252.97 |
| 515F-806R | *V. velutina*1 | 35239 | 8914109 | 252.96 |
| 515F-806R | *V. velutina*2 | 38176 | 9656769 | 252.95 |
| 515F-806R | *V. velutina*3 | 35095 | 8868903 | 252.71 |
| 515F-806R | *V. bicolor*1 | 33238 | 8407970 | 252.96 |
| 515F-806R | *V. bicolor*2 | 35678 | 9022731 | 252.89 |
| 515F-806R | *V. bicolor*3 | 35850 | 9057885 | 252.66 |

**Table S6.** Comparison of PICRUSt2-based microbial function prediction (level_1)

| KEGG_L1 | *V.tropica* | *V.basalis* | *V.velutina* | *V.bicolor* |
| --- | --- | --- | --- | --- |
| Cellular Processes | 3.80% | 3.42% | 2.57% | 2.47% |
| Environmental Information Processing | 10.82% | 11.16% | 10.24% | 10.43% |
| Genetic Information Processing | 14.10% | 14.57% | 17.43% | 17.51% |
| Human Diseases | 1.69% | 1.58% | 1.38% | 1.34% |
| Metabolism | 68.61% | 68.39% | 67.77% | 67.71% |
| Organismal Systems | 0.96% | 0.89% | 0.61% | 0.54% |

Note: Values represent the relative abundance (%) of predicted KEGG Level 1 functional categories inferred from 16S rRNA gene sequences **of four *Vespa* species** using PICRUSt2.

**Table S7.** Comparison of PICRUSt2-based microbial function prediction (level_2)

| KEGG_L2 | *V. tropica* | *V. basalis* | *V.velutia* | *V. bicolor* |
| --- | --- | --- | --- | --- |
| Amino acid metabolism | 13.63% | 13.21% | 12.31% | 11.97% |
| Biosynthesis of other secondary metabolites | 1.30% | 1.22% | 0.97% | 0.99% |
| Cancer: overview | 0.16% | 0.14% | 0.11% | 0.10% |
| Cancer: specific types | 0.23% | 0.20% | 0.15% | 0.13% |
| Carbohydrate metabolism | 16.10% | 16.25% | 16.77% | 17.03% |
| Cardiovascular disease | 0.05% | 0.05% | 0.03% | 0.02% |
| Cell growth and death | 1.09% | 1.04% | 1.08% | 1.06% |
| Cell motility | 2.18% | 1.86% | 1.07% | 1.01% |
| Cellular community - eukaryotes | 0.00% | 0.00% | 5.55% | 0.00% |
| Cellular community - prokaryotes | 0.13% | 0.14% | 0.14% | 0.14% |
| Circulatory system | 0.09% | 0.06% | 0.03% | 0.02% |
| Development and regeneration | 0.00% | 0.00% | 5.34% | 0.00% |
| Digestive system | 0.04% | 0.03% | 0.02% | 0.01% |
| Drug resistance: antimicrobial | 0.01% | 0.01% | 0.01% | 0.01% |
| Endocrine and metabolic disease | 0.15% | 0.16% | 0.20% | 0.20% |
| Endocrine system | 0.41% | 0.41% | 0.27% | 0.23% |
| Energy metabolism | 8.19% | 8.06% | 7.60% | 7.53% |
| Environmental adaptation | 0.26% | 0.25% | 0.22% | 0.21% |
| Excretory system | 0.05% | 0.05% | 0.03% | 0.03% |
| Folding, sorting and degradation | 2.41% | 2.34% | 2.38% | 2.35% |
| Glycan biosynthesis and metabolism | 2.16% | 2.19% | 2.24% | 2.28% |
| Immune disease | 0.09% | 0.10% | 0.11% | 0.12% |
| Immune system | 0.11% | 0.09% | 0.05% | 0.04% |
| Infectious disease: bacterial | 0.16% | 0.21% | 0.35% | 0.39% |
| Infectious disease: parasitic | 0.12% | 0.11% | 0.06% | 0.05% |
| Lipid metabolism | 4.44% | 4.45% | 4.25% | 4.22% |
| Membrane transport | 7.48% | 7.75% | 7.33% | 7.44% |
| Metabolism of cofactors and vitamins | 6.44% | 6.23% | 5.71% | 5.78% |
| Metabolism of other amino acids | 3.26% | 3.24% | 3.15% | 3.12% |
| Metabolism of terpenoids and polyketides | 2.20% | 2.23% | 2.18% | 2.18% |
| Nervous system | 0.00% | 0.00% | 0.00% | 2.31% |
| Neurodegenerative disease | 0.72% | 0.60% | 0.37% | 0.31% |
| Nucleotide metabolism | 6.78% | 7.37% | 9.09% | 9.27% |
| Replication and repair | 5.25% | 5.47% | 6.61% | 6.67% |
| Signal transduction | 3.34% | 3.41% | 2.91% | 2.99% |
| Transcription | 0.80% | 0.77% | 0.80% | 0.78% |
| Translation | 5.64% | 5.98% | 7.64% | 7.71% |

**Table S7 (continued).** Comparison of PICRUSt2-based microbial function prediction (level_2)

| KEGG_L2 | *V. tropica* | *V. basalis* | *V.velutia* | *V. bicolor* |
| --- | --- | --- | --- | --- |
| Transport and catabolism | 0.41% | 0.38% | 0.28% | 0.26% |
| Xenobiotics biodegradation and metabolism | 4.10% | 3.94% | 3.50% | 3.34% |

Note: Values represent the relative abundance (%) of predicted KEGG Level 2 functional categories inferred from 16S rRNA gene sequences **of four *Vespa* species** using PICRUSt2.
